# Genomic and Functional Characterization of Novel Phages Targeting Multidrug-Resistant *Acinetobacter baumannii*

**DOI:** 10.3390/ijms26136141

**Published:** 2025-06-26

**Authors:** Alma Karen Orozco-Ochoa, Beatriz Quiñones, Jean Pierre González-Gómez, Nohelia Castro-del Campo, José Benigno Valdez-Torres, Cristóbal Chaidez-Quiroz

**Affiliations:** 1 Laboratorio Nacional para la Investigación en Inocuidad Alimentaria (LANIIA), Centro de Investigación en Alimentación y Desarrollo, A.C. (CIAD), Carretera a Eldorado Km 5.5, Campo El Diez, Culiacán 80110, Sinaloa, Mexico; aorozco222@estudiantes.ciad.mx (A.K.O.-O.); jean.gonzalez@ciad.mx (J.P.G.-G.); ncastro@ciad.mx (N.C.-d.C.); jvaldez@ciad.mx (J.B.V.-T.); 2 Produce Safety and Microbiology Research Unit, Western Regional Research Center, Agricultural Research Service, U.S. Department of Agriculture, Albany, CA 94710, USA; beatriz.quinones@usda.gov

**Keywords:** multidrug-resistant *Acinetobacter baumannii*, antimicrobial resistance, phages, alternative intervention strategies, phage characterization

## Abstract

*Acinetobacter baumannii* is an opportunistic pathogen and a major cause of nosocomial infections worldwide. This study aimed to isolate and characterize phages with lytic activity against multidrug-resistant *A. baumannii* strains to enable antibacterial alternatives. Eight phages (AKO8a, PS118, B612, MCR, IDQ7, 89P13, CRL20, and CIM23) were isolated and subjected to genomic, phylogenetic, and functional analyses. Antibacterial activity was assessed in vitro against *A. baumannii* strain AbAK04 by measuring optical density over 17 h at multiplicities of infection (MOIs) of 0.1, 1, and 10, using a repeated-measures design with time as a crossed factor and MOI as a nested factor. Tukey’s post-hoc test identified significant bacterial growth reductions of 57–72% (*p* < 0.001). Specifically, phages PS118 and 89P13 reduced growth by 71% at MOI 10; CIM23, B612, and CRL20 achieved 68% reduction at MOI 1; and MCR reduced growth by 64% at MOIs 0.1 and 1. Notably, lytic phage MCR encodes a glycosyl hydrolase family 58 (GH58) enzyme, potentially contributing to its antibacterial activity. Genomic analyses confirmed absence of virulence and antibiotic resistance genes, with all phages classified as novel species within the *Kagunavirus* genus. These findings support the use of these phages as promising candidates for in vivo evaluation.

## 1. Introduction

The advent of antibiotics revolutionized medicine, but their misuse and overuse have accelerated the emergence of antimicrobial resistance (AMR), a major global health concern [1,2]. AMR is now ranked among the top ten global health threats [3], with projections of 10 million deaths annually by 2050 [4,5]. *Acinetobacter baumannii*, a top-priority pathogen included in the ESKAPE group (*Enterococcus faecium*, *Staphylococcus aureus*, *Klebsiella pneumoniae*, *Acinetobacter baumannii*, *Pseudomonas aeruginosa*, and *Enterobacter* spp.) [6,7,8], has been implicated in hospital-acquired infections, rapid resistance gene acquisition, and rising carbapenem resistance worldwide [9]. In Mexico, *A. baumannii* was the fourth most isolated pathogen in 2023, with 550 healthcare-associated infections [10]. Specifically, in the state of Sinaloa in Northwestern Mexico, *A. baumannii* was identified as the first most common causative agent in primary healthcare infections, comprising 13.48% of total reported cases in January 2025 [11].

The use of phages has become a promising alternative to combat AMR, particularly against *A. baumannii*. Phages were discovered around the same time as antibiotics. Phages specifically target and kill bacteria [12]. Phage-based intervention reports have recently increased, reflecting rapid progress in translating this technology from laboratory study to clinical application in patients [13]. However, challenges remain in the development, acceptance, and approval of phages for widespread clinical use [14,15,16]. To ensure treatment viability, phages must undergo rigorous characterization before application [12]. In particular, phages can follow either a lytic cycle (virulent phages) or a lysogenic cycle (temperate phages). In the lytic cycle, phages replicate and lyse the host cell to release new virions [17]. In the lysogenic cycle, phages integrate into the host genome as prophages, remaining dormant until reactivation triggers the lytic cycle [18,19]. The full potential of temperate phages as antimicrobial agents in combating AMR has been investigated since these phages are easier to isolate and are distinct mechanistically in the synergy between antibiotics and the phages [20,21,22,23]. However, their therapeutic use is controversial due to risks of lysogeny and horizontal gene transfer [24], requiring thorough genomic and phenotypic characterization essential to ensure safety [25], such as testing for spontaneous phage release, especially when sequencing data are limited [26].

Only a few phages capable of infecting *A. baumannii* have been identified, including both lytic and temperate phages such as 5W, ABMM1, ΦFG02, and ΦCO01 [12,27,28]. Notably, the temperate phage ABMM1 effectively kills *A. baumannii* at a high multiplicity of infection and reduces infection severity in a zebrafish model, demonstrating its potential as an alternative treatment [27]. Lytic phage ΦFG02 drives *A. baumannii* AB900 to evolve into a phage-resistant, capsule-deficient form that resensitizes to ceftazidime, illustrating phage therapy’s potential to restore antibiotic effectiveness [29]. However, these documented phages are limited by factors such as host range, stability, and bacterial defense evasion. Similarly, Bagińska et al. [30] isolated twelve *A. baumannii* phages, eleven of which were temperate, and only one was lytic. Their study did not include a comprehensive analysis of the phages’ genetic composition nor assess their therapeutic potential in vivo, emphasizing the need for further research in these areas. Phage Indie, on the other hand, reduces *A. baumannii* resistance to ceftazidime, enhancing its activity and demonstrating its promise as an antimicrobial agent in phage–antibiotic synergy in vitro [31].

Given the growing concern over AMR and the urgent need for alternative treatments due to the limited effectiveness of current intervention alternatives against multidrug-resistant *A. baumannii*, the present study aimed to isolate and conduct a genomic and functional characterization of the recovered phages to further assess their safety as alternative antimicrobial agents. These findings documented the development of a well-characterized phage library targeting clinical bacterial isolates and provided enabling approaches for future in vivo trials in conjunction with the effective use of phages against multidrug-resistant bacterial pathogens.

## 2. Results

### 2.1. Antimicrobial Susceptibility Testing

**Figure 1:**
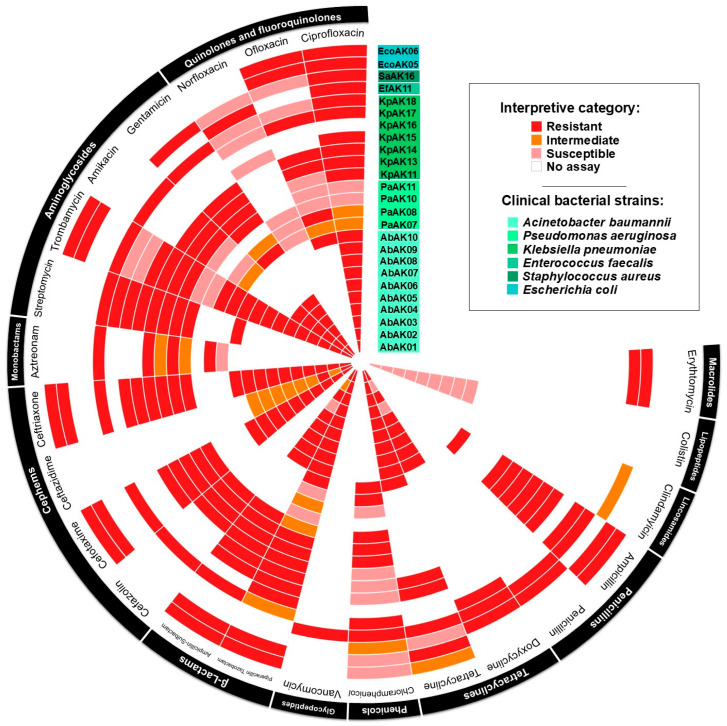
Antimicrobial resistance pattern of clinical strains of *Acinetobacter baumannii*, other ESKAPE pathogens, and Gram-negative pathogens. The antimicrobial resistance profiles were determined by measuring the diameter of the inhibition zone using the Kirby–Bauer disk diffusion assay. The tested antimicrobials were specific to each bacterial genus according to CLSI protocols.

All tested clinical isolates exhibited multidrug resistance, defined as resistance to three or more antimicrobial classes. Resistance profiles varied across species but were particularly pronounced in *A. baumannii* strains, which consistently resisted β-lactams, aminoglycosides, and fluoroquinolones [8]. A summary of the resistance patterns for all tested strains is presented in Figure 1.

### 2.2. Phage Isolation, Plaque Characterization, and Host Range Analysis

**Figure 2:**
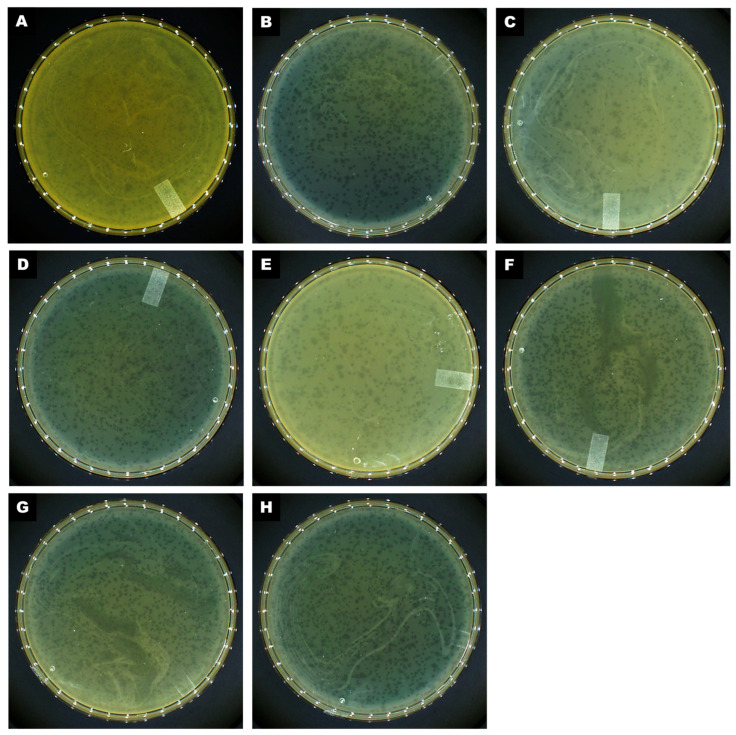
Morphology of plaques formed on a double-layered agar plate by (**A**) CRL20, (**B**) 89P13, (**C**) AKO8a, (**D**) PS118, (**E**) IDQ7, (**F**) MCR, (**G**) CIM23, and (**H**) B612.

From the analysis of sixteen wastewater samples collected from treatment plants in Sinaloa, Mexico, the initial objective was to isolate phages active against members of the ESKAPE group of pathogens. A total of twenty-two phages were isolated, all showing lytic activity against multidrug-resistant *A. baumannii*. All phages produced clear plaques of varying diameters on trypticase soy agar plates. Host range analysis using ten clinical *A. baumannii* strains, thirteen ESKAPE pathogens (*P. aeruginosa*, *K. pneumoniae*, *E. faecalis*, *S. aureus*), and two Gram-negative pathogens (*E. coli*) confirmed that the recovered phages exclusively infect their host bacterium, *A. baumannii* strain AbAK04 (Table 1). Among them, eight phages exhibited high infective production and plating efficiency on *A. baumannii* strain AbAK04, with titers ranging from 6.0 × 10^8^ to 1.0 × 10^10^ plaque-forming units (PFU)/mL, making them suitable candidates for evaluating their antibacterial potential (Figure 2).

### 2.3. Comparative Genomics and Phylogenetic Analysis of Acinetobacter baumannii-Targeting Phages as a Promising Alternative Intervention

Genome sequencing of the eight selected phages (AKO8a, PS118, B612, MCR, IDQ7, 89P13, CRL20, and CIM23) was performed for further characterization of the phages, and a temperate lifestyle was confirmed with PhageAI version 0.10.0 software. The bioinformatic analysis revealed that the phages’ genetic material consisted of double-stranded DNA and exhibited variations in genome length, GC content, and number of open reading frames (ORFs) (Table 2). Noteworthy, no virulence or antibiotic resistance genes were detected in any of the phage genomes through searches of the ABRIcate databases, and no tRNA genes were identified using tRNAscan-SE version 2.0 software.

The number of ORFs with similarity to known genes varied among the eight examined phages. Phage AKO8a had the highest proportion, with 149 ORFs matching known function genes and only eight predicted as hypothetical proteins. In contrast, the remaining phages had between 34 and 39 ORFs associated with known functions (Table 2). The number of hypothetical proteins ranged from 105 in phage MCR to 123 in PS118, with most phages carrying over 100 hypothetical ORFs. Predicted ORFs in the phage genomes exhibited between 98% and 100% similarity with *Escherichia coli* phages, such as vB_EcoS_XY1 (YP_010749717.1), JSSK01 (WFD55412.1), vB_EcoS-phiEc3 (YP_010749508.1), K1ind2 (YP_009597365.1), and K1H (YP_009168857.1).

**Figure 3:**
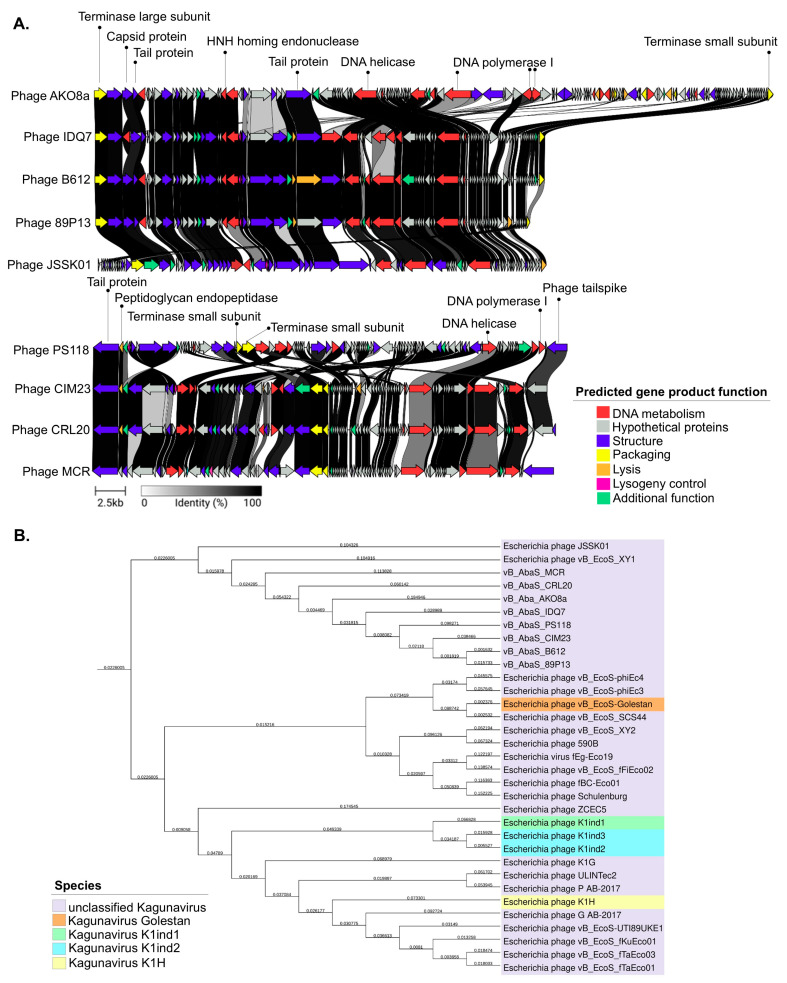
Genomic characterization of phages within the *Kagunavirus* genus. (**A**): Phage comparison with the phage JSSK01 reference genome performed using the Clinker tool. Homologous regions between phages are shown with gray shading. Colored arrows represent ORFs based on their predicted function according to their module. (**B**): Phylogenetic tree of phages constructed using Genome-BLAST Distance Phylogeny within the *Kagunavirus* genus, *Guernseyvirinae* subfamily.

All predicted ORFs were clustered into seven functional modules: DNA metabolism, hypothetical proteins, structure, packaging, lysogeny control, and additional functions, as shown in Figure 3A. Interestingly, genomic maps revealed a putative immunity protein, QCF69_gp43 associated with lysogeny in five phages (PS118, B612, IDQ7, 89P13, CIM23) (Figures S1 and S2). By contrast, the genomes of phages MCR, AKO8a, and CRL20 lacked any lysogeny-related genes. The analysis also demonstrated that all phage genomes encoded proteins for DNA metabolism (e.g., DNA polymerase, helicase, HNH endonuclease, adenine-specific methyltransferase), structural components (e.g., tail fiber, major capsid, minor tail, and assembly chaperones), and genome packaging (small and large terminase subunits). Related to lysis functions, all phages encoded lysozyme, endopeptidase, endolysin, holin and o-spanin. Of the eight phages analyzed, only phage MCR encoded a glycosyl hydrolase family 58 (GH58) corresponding to ORF62, which contains N-terminal domains of the phage endosialidase (pfam PF12218), and a catalytic beta propeller domain of phage endosialidase (pfam PF12217).

**Figure 4:**
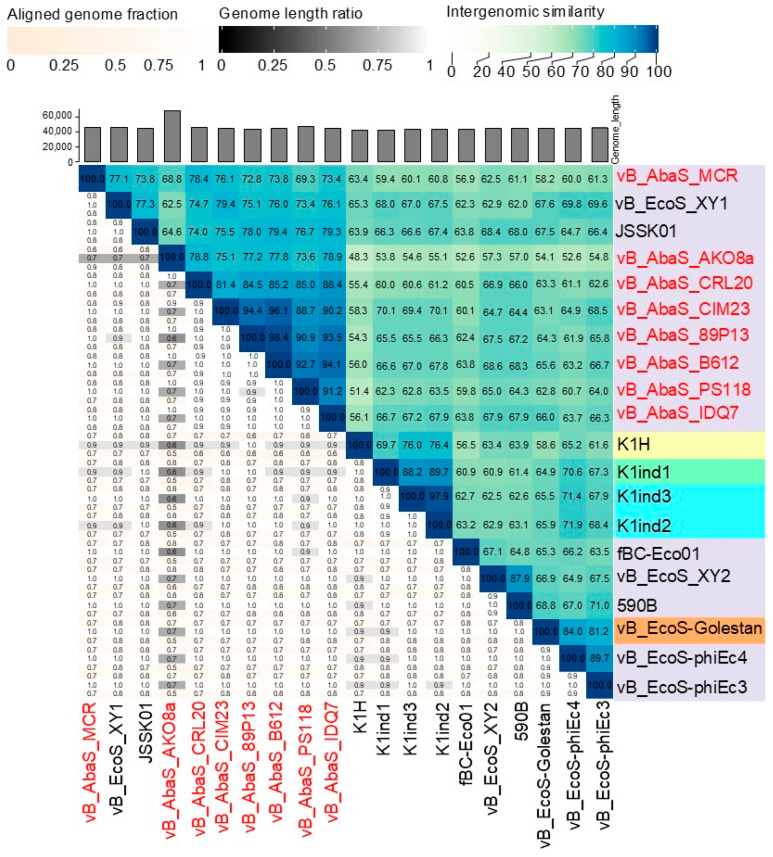
Taxonomic assignment of *Acinetobacter* phages through comparative genomics. Heatmap of VIRIDIC software. The values of percentage identity range from 0 (0%, white) to 1 (100%, blue).

Subsequent BLASTN nucleotide analysis of the phage genomes revealed a close sequence similarity to 12 *E. coli* phages, with more than 80% nucleotide identity to the eight selected phages. Phage JSSK01 (OQ442786.1) was identified as the closest relative, showing more than 80% coverage and more than 95% identity. The phylogenetic tree confirmed that *E. coli* phages were potentially the closest ancestors (Figure 3B). Intergenomic comparisons using VIRIDIC software (available online: https://rhea.icbm.uni-oldenburg.de/viridic/; accessed on 8 April 2025) (Figure 4) placed all eight phages within the genus *Kagunavirus*, with more than 70% similarity to *E. coli* phages JSSK01 and vB_EcoS_XY1, suggesting that the phages examined in the present study represent a novel, unclassified species. Following the International Committee on Taxonomy of Viruses (ICTV) criteria, the phages were designated as *A. baumannii* phages vB_AbaS_AKO8a, vB_AbaS_PS118, vB_AbaS_B612, vB_AbaS_MCR, vB_AbaS_IDQ7, vB_AbaS_89P13, vB_AbaS_CRL20, and vB_AbaS_CIM23. Phage common names are AKO8a, PS118, B612, MCR, IDQ7, 89P13, CRL20, and CIM23, respectively. Classification was confirmed using PhageAI version 0.10.0 software with a 99.2% probability, aligning with ICTV taxonomy.

### 2.4. Evaluating Lysogenic Activity in Phages

Based on the bioinformatics analysis, the temperate lifestyle of the recovered phages was further examined by using the genome-based predictions to obtain evidence of the phage’s replication strategy. To further characterize their ability to induce rapid infections in the target bacterial strains, the phage lifestyle was validated using lysogenicity assays. In particular, these assays determined whether a phage integrated into the host genome, indicating a lysogenic lifestyle [26]. Alternatively, the phages were assessed for following a strictly lytic cycle, which involves immediate replication, production of new phage particles, and lysis of the host cell without integration [32].

The assay results demonstrated that phages PS118, B612, IDQ7, 89P13, and CIM23 did not form “mesas” at the tested concentrations (10^4^, 10^6^, and 10^8^) over 96 h of co-incubation with *A. baumannii* strain AbAK04, indicating an inability to lysogenize the host under the tested conditions (Figure S3). This suggests that these phages were unable to establish lysogeny. In contrast, phages AKO8a, CRL20, and MCR formed “mesas” at a concentration of 10^4^ after 96 h of co-incubation with *A. baumannii* strain AbAK04 (Figure S4A). Subsequent patch plate assays revealed that colonies of *A. baumannii* strain AbAK04 appeared to harbor prophages (MCR-17, AKO8a-9, AKO8a-10, AKO8a-11, CRL20-3, CRL20-4, CRL20-5, CRL20-6, CRL20-7). Even after repeating this assay multiple times, the *A. baumannii* colonies showed no signs of any spontaneous lysis (Figure S4B).

To confirm that the host colonies did not harbor integrated prophages, we performed additional confirmatory analyses (Figure S4C,D), such as spot tests with culture supernatants as well as homoimmunity assays. Both types of assays yielded negative results indicative of the absence of lysogeny because no lysis zones were observed in the supernatant spot test, suggesting a lack of spontaneous phage release. Also, no protection from superinfection was detected in the homoimmunity assay, ruling out the presence of functional prophages. In summary, these results collectively confirm that phages AKO8a, CRL20, and MCR do not establish lysogeny in *A. baumannii* strain AbAK04 under the tested conditions, and instead, the examined phages follow a strictly lytic replication cycle.

### 2.5. Antibacterial Activity, One-Step Growth Curve, and Phage Adsorption Rate

**Figure 5:**
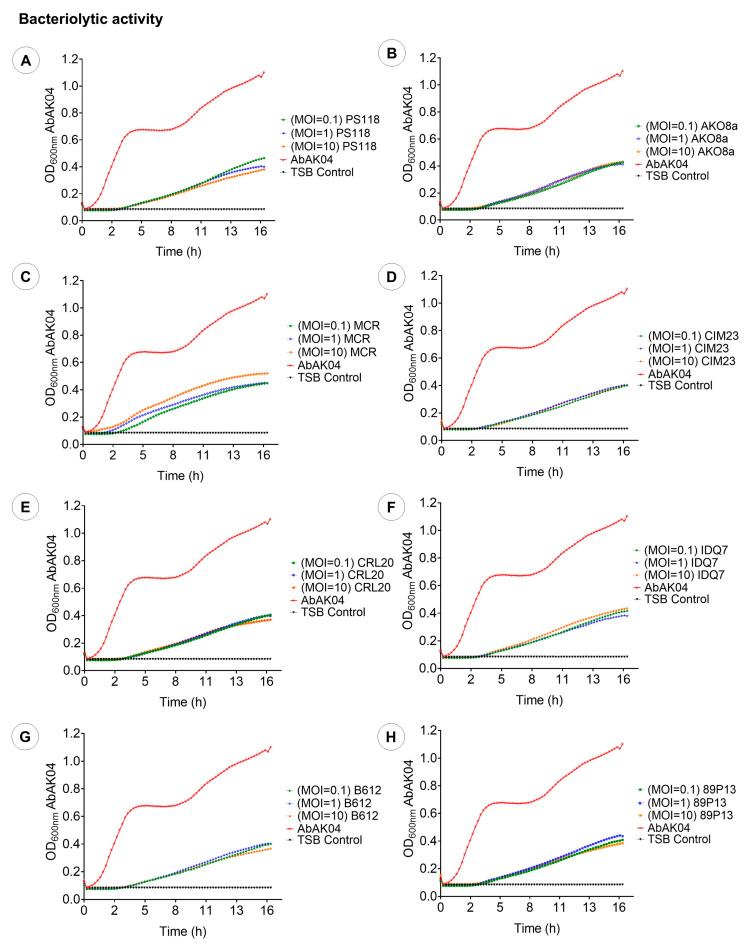
Bacteriolytic activity of eight phages at various MOIs (10, 1, and 0.1). (**A**): PS118. (**B**): AKO8a. (**C**): MCR. (**D**): CIM23. (**E**): CRL20. (**F**): IDQ7. (**G**): B612. (**H**): 89P13. Each graph presents the mean values of three tests (n = 3) ± standard deviation (SD), error bars represent the standard deviation.

After 17 h of co-incubation with *A. baumannii* strain AbAK04, the evaluated phage MOI values significantly reduced bacterial growth in liquid culture, as determined by absorbance measurements, with reductions ranging from 57% to 72% (Figure 5). The repeated-measures design indicated that there were significant differences (*p* < 0.001) in bacterial growth among the different MOIs. Specifically, phages PS118 (Figure 5A) and 89P13 (Figure 5H) reduced bacterial levels by 71% at MOI 10. Phages CIM23 (Figure 5D), B612 (Figure 5G), and CRL20 (Figure 5E) achieved a 68% reduction at MOI 1. Meanwhile, phage MCR (Figure 5C) reduced the bacterial population by 64% at MOIs of 0.1 and 1. In summary, all of these results confirm the antibacterial activity of the examined phages.

**Figure 6:**
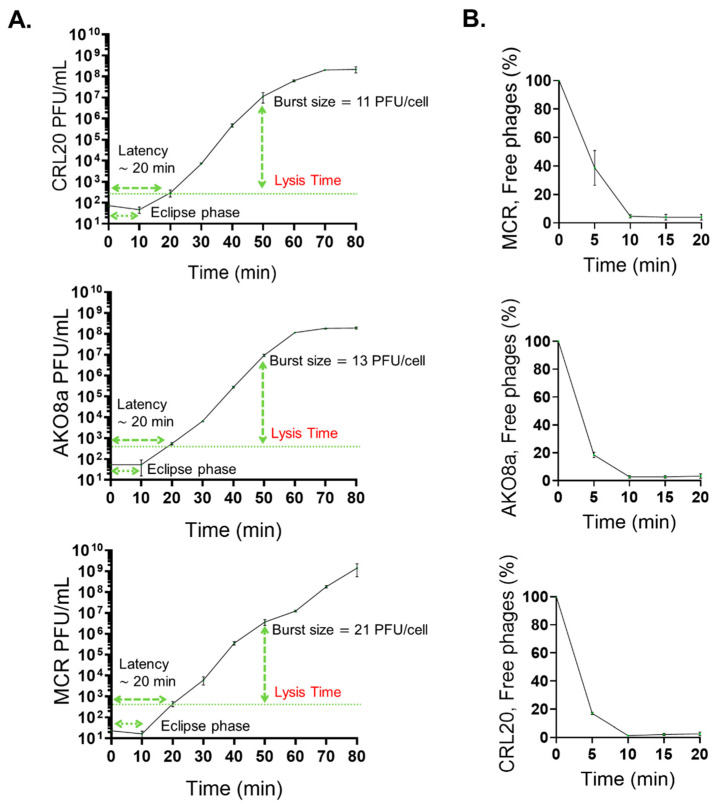
Lytic activity assays. (**A**): One-step growth curve for lytic phages AKO8a, MCR, and CRL20. The latency time corresponds to the time before newly formed phages are first detected in the supernatant. The lysis time, however, is defined as the time point at which phage release reaches a plateau, reflecting the completion of cell lysis in the majority of the population. Burst size was calculated at this plateau. (**B**): Adsorption curve for lytic phages AKO8a, MCR, and CRL20. Each graph presents the mean values of three tests (n = 3) ± standard deviation (SD).

Following the growth reduction assays, the next experimental approach aimed to investigate the replication dynamics of the phages, focusing on their latency period, burst size, and adsorption rate to determine how efficiently the examined phages replicated and interacted with the bacterial host. The results from these assays demonstrated that the lysogenic phages PS118, B612, IDQ7, 89P13, and CIM23, which encoded the putative immunity protein QCF69_gp43, did not replicate after three trials, and no latency period, burst size, or adsorption rate results were obtained. In contrast, the lytic phages CRL20, AKO8a, and MCR exhibited a latency period of 20 min, with burst sizes of 11, 13, and 21 PFU/cell, respectively (Figure 6A). Notably, the burst size was calculated based on the total number of phages released at the plateau stage of the one-step growth curve (lysis time ~50 min), since not all cells lyse synchronously immediately after the latency period. Adsorption assays showed that 60% of CRL20, 80% of AKO8a, and 61% of MCR phages adhered to *A. baumannii* strain AbAK04 within 5 min (Figure 6B).

## 3. Discussion

Phages have gained significant interest in recent years for their potential as antimicrobial agents against multidrug-resistant pathogens, offering a promising alternative to combat antimicrobial resistance [33,34,35]. Phages are seen as a promising solution to combat *A. baumannii* [9,29,36], currently the most critical nosocomial pathogen [7,8]. However, isolating virulent *A. baumannii* phages remains a significant challenge. The bacterium employs a variety of defense mechanisms to evade phage infection, including inhibition of phage adsorption, CRISPR-Cas systems, restriction-modification systems, and superinfection immunity, all of which hinder successful phage infection and replication [37,38,39]. These challenges are combined by extensive genetic diversity and genetic mosaicism among phages, which complicates the identification of lytic candidates with antibacterial activity [40,41]. Therefore, most recent studies have reported the isolation of temperate and lytic phages as alternative antimicrobial agents against *A. baumannii* [12,27,28,30,42]. To address this limitation, establishing large, well-characterized phage libraries has been proposed as a viable strategy [41]. Phages with clinical potential should undergo thorough genomic and phenotypic characterization and be deposited in centralized phage banks accessible to the global medical community to support the development of effective phage-based treatments.

Given the urgent need to isolate and characterize novel phages with antimicrobial potential [43], the present study conducted a screen of phages from wastewater infecting multidrug-resistant *A. baumannii*. By targeting specifically multidrug-resistant *A. baumannii*, a total of eight phages were subsequently selected for further characterization with the ultimate goal of enabling a highly specific therapeutic approach by minimizing unintended effects on beneficial microbiota and reducing the risk of resistance selection in other bacterial species. The specificity of these eight phages could be particularly advantageous for personalized phage therapy, where tailored treatments can be designed based on the infecting strain, optimizing efficacy while avoiding collateral impacts on the patient’s microbiome [44]. Currently, there is no standardized criterion for defining broad versus narrow host ranges [43,45]. While phages in natural environments, such as wastewater, coevolve with bacteria and may gain the ability to infect multiple strains or genera [46], the precision of phages with a very specific host range offers a targeted and controlled strategy for managing infections, particularly in the context of multidrug-resistant pathogens, as is *A. baumannii*.

The genomic classification of the examined eight phages as a new species within the *Kagunavirus* genus was justified by their nucleotide identity being less than 95% compared to the reference phages, supporting their classification based on established species delimitation criteria for phages [47]. While comprehensive, future studies incorporating transmission electron microscopy (TEM) imaging would further confirm and expand the biological characterization of these phages. Moreover, the phage genomic analysis, conducted in the present study, revealed an absence of antibiotic resistance and virulent genes, suggesting that the recovered phages could serve as a promising antimicrobial alternative highlighting their potential as a safe and effective solution against multidrug-resistant *A. baumannii* [16]. Additionally, the observed absence of tRNA genes indicated phage dependence on the host bacteria for protein synthesis [48].

Based on ORF classification, the recovered phages in the present study were found to encode genes associated with several functional modules, highlighting their potential antibacterial activity. Lytic enzymes were identified as key factors in bacterial elimination [31]. Among them, lysozymes play a crucial role by lysing host cells to release phage particles [49], along with endolysins and endopeptidases that degrade bacterial peptidoglycan [50]. Genes encoding holins, membrane proteins responsible for disrupting the bacterial membrane, and spanins, which facilitate outer membrane lysis, were also detected [19,51]. The DNA metabolism module included proteins with helix-turn-helix domains for DNA binding [52] and adenine-specific methyltransferases, which regulate gene expression via DNA methylation [53]. HNH endonucleases, located near terminase genes, were present and are involved in DNA packaging and homologous recombination [54]. Additionally, transcriptional regulators encoded by these temperate phages may trigger the lytic cycle in response to DNA damage [55], potentially influencing bacterial processes such as virulence, metabolism, antibiotic resistance, and stress responses [56].

Of particular interest, this study revealed that the five temperate phages (PS118, B612, IDQ7, 89P13, and CIM23) carried a superinfection immunity protein, QCF69_gp43, which may play a role in regulating phage–phage interactions [9]. Superinfection immunity is a mechanism that allows a prophage-infected bacterium to resist subsequent infections by other phages, typically through repressor proteins that block the replication of invading phages [57,58,59]. While such mechanisms can be beneficial for the long-term persistence of phages and may help prevent secondary phage infections in chronic scenarios, temperate phages are not suitable for therapeutic use under current clinical guidelines [60,61]. This is due to their unpredictable induction into the lytic cycle [62], inability to rapidly lyse host bacteria, and, above all, their potential to mediate horizontal gene transfer [24], including virulence or resistance determinants, to otherwise avirulent bacterial populations [20]. Although the temperate phages described in this study lack detectable antibiotic resistance or virulence genes and do not carry key lysogeny-related genes (e.g., integrases, transposases, repressors) [57,63,64], their use as therapeutic agents is not recommended unless these phages are genetically engineered to eliminate the lysogeny-associated superinfection immunity protein QCF69_gp43 prior to any in vivo application as an antibacterial agent.

The antimicrobial activity of phages AKO8a, PS118, B612, MCR, IDQ7, 89P13, CRL20, and CIM23 was confirmed through in vitro bacteriolytic assays, with some showing comparable lytic efficacy to previously characterized phages such as vB_AbaM_ABMM [27], Abp95 [65], Abgy202141 [66], and vAbaIN10 [67]. Notably, five of these phages (PS118, B612, IDQ7, 89P13, and CIM23) harbor genes associated with lysogeny, yet were still able to significantly reduce bacterial populations, suggesting their potential as antimicrobial agents. Further investigation into possible genetic modification could enhance their suitability for phage-based therapeutic strategies.

The genome analysis for phages AKO8a, MCR, and CRL20 revealed a lack of lysogeny-associated genes, classifying them as lytic phages with potential as antimicrobial agents against multidrug-resistant *A. baumannii*. One-step growth curves and adsorption data for the lytic phages AKO8a, MCR, and CRL20 revealed smaller burst sizes and shorter latency periods compared to the temperate phage øCO01, but similar to the lytic phage øFG02 [12]. Phage AKO8a had an adsorption period comparable to vB_AbaM_ABMM1 [27], while phages MCR and CRL20 showed approximately 30% lower adsorption rates than other *Acinetobacter* phages [68]. The slower adsorption rates observed for MCR and CRL20 may suggest distinct mechanisms of bacterial recognition and infection [69]. These findings underscore the potential of phages with diverse adsorption kinetics to enhance therapeutic approaches, positioning them as promising candidates for future in vivo assays. Interestingly, the presence of a glycosyl hydrolase family 58 (GH58) enzyme in the phage MCR suggests that it may function as an endosialidase, facilitating phage infection by degrading the bacterial capsule. This enzyme is commonly found in phages that infect bacteria with polysialic acid capsules, such as *E. coli* K1 phages [70]. *A. baumannii* displays a wide variety of capsule types (K loci), responsible for producing distinct polysaccharides, some potentially containing sialic acid-like components [71,72]. Further research and experimental validation are thus necessary to clarify how the tailspike proteins of phage MCR recognize and degrade these surface structures, such as in the phage ABPH49 [73].

In conclusion, the phages characterized in this study, particularly the three lytic phages MCR, AKO8a, and CRL20, demonstrate potential as biological antimicrobial agents, warranting future in vivo assays. This work contributes to expanding intervention libraries against multidrug-resistant *A. baumannii*, a globally critical pathogen, and represents an important step toward the standardization of phage therapy as an antimicrobial strategy worldwide.

## 4. Materials and Methods

### 4.1. Bacterial Strains, Culture Conditions, and Storage

The ten clinical *A. baumannii* strains, thirteen ESKAPE pathogens (*P. aeruginosa*, *K. pneumoniae*, *E. faecalis*, and *S. aureus*), and two Gram-negative pathogens (*E. coli*) used in this study were obtained from the strain collection at a public hospital in Culiacán, Sinaloa, Mexico. The bacterial identity of each clinical isolate was confirmed through Gram-staining and Vitek^®^2 (bioMérieux, Durham, NC, USA). An endpoint polymerase chain reaction (PCR) was performed to confirm the species of the bacterial host of the phages, using species-specific primers for *A. baumannii* [74], F: 5′-TAATGCTTTGATCGGCCTTG-3′ and R: 5′-TGGATTGCACTTCATCTTGG-3′; for *P. aeruginosa* [75], F: 5′-CTGGGTCGAAAGGTGGTTGTTATC-3′ and R: 5′-GCGGCTGGTGCGGCTGAGTC-3′; for *E. faecium* [76], F: 5′-GAAAAAACAATAGAAGAATTAT-3′ and R: 5′-TGCTTTTTTGAATTCTTCTTTA-3′; for *S. aureus* [77], F: 5′-GCGATTGATGGTGATACGGTT-3′ and R: 5′-AGCCAAGCCTTGACGAACTAAAGC-3′; for *K. pneumoniae* [78], F: 5′-CAACCATGGTGGTCGATTAG-3′ and R: 5′-TGGTAGCCATATCCCTTTGG-3′; and for *E. coli* [79]. The amplified products were analyzed using 1% agarose gels supplemented with 0.04 μL/mL GelRed nucleic acid stain (Phoenix Research, Candler, NC, USA), and the nucleotide sequences of the amplicons were confirmed through conventional Sanger DNA sequencing (Elim Biopharmaceuticals, Inc., Hayward, CA, USA). All bacterial strains were propagated in tryptic soy broth (TSB; Oxoid Ltd., Hants, UK) at 37 °C for 24 h and were stored at −80 °C in 20% (*v*/*v*) glycerol.

### 4.2. Antimicrobial Susceptibility Profile

The antimicrobial resistance in the ten clinical *A. baumannii* strains, thirteen ESKAPE pathogens, and two Gram-negative pathogens was performed using the disk diffusion method according to Clinical and Laboratory Standards Institute guidelines (CLSI) [80], with minor modifications. The Kirby–Bauer method was applied on Mueller Hinton agar plates for each tested bacterial isolate. Twenty-three antibiotics, classified into twelve distinct classes, were tested with an appropriate disk (Oxoid Ltd., Basingstoke, UK) at the following amounts: ciprofloxacin (CIP, 5 μg), ofloxacin (OFX, 5 μg), norfloxacin (NOR, 10 μg) [Quinolones and fluoroquinolones], gentamicin (GM, 10 μg), amikacin (AN, 30 μg), tobramycin (NN, 10 μg), streptomycin (S, 10 μg) [Aminoglycosides], aztreonam (ATM, 30 μg) [Monobactams], ceftriaxone (CRO, 30 μg), cefotaxime (CTX, 30 μg), cefozolin (CZ, 30 μg), ceftazidime (CFZ, 30 μg) [Cephems], ampicillin-sulbactam (SAM, 10/10 μg), piperacillin-tazobactam (TZP, 100/10) [β-Lactams], vancomycin (VAN, 30 μg) [Glycopeptides], chloramphenicol (C, 30 μg) [Phenicols], tetracycline (TE, 30 μg), doxycycline (D, 30 μg) [Tetracyclines], penicillin (P, 10 μg), ampicillin (AM, 10 μg) [Penicillins], clindamicina (CC, 2 μg) [Lincosamides], colistin (CT) [Lipopeptides], erythromicyn (E, 15 μg) [Macrolides]. *Escherichia coli* strain ATCC 25922 was employed as positive control for all experiments, each consisting of three replicates per condition tested.

### 4.3. Phage Isolation, Propagation, and Titration

To investigate the potential of phages against ESKAPE pathogens, initial efforts were focused on *A. baumannii,* a clinically relevant member of this group and a critical-priority multidrug-resistant pathogen [7,8]. Phages were isolated using *A. baumannii* as the bacterial host to facilitate the enrichment and recovery of lytic phages from environmental sources. A total of 16 samples of hospital sewage and wastewater were collected for this purpose. For the phage identification method, hospital wastewater samples were pretreated before being used in the phage propagation assay, following the procedures described previously [81]. Bacterial cultures were prepared using a previously described enrichment [48] method with some modifications. Briefly, 1 mL of wastewater was mixed with 30 mL of double-strength TSB (Oxoid Ltd., Hants, UK) and 1 mL of bacterial culture. The mixture was then incubated at 37 °C for 18 h with gentle shaking at 50 rpm. After incubation, the mixture was centrifuged at 8000× *g* for 10 min at 4 °C using a Megafuge16R (Thermo Fisher Scientific Inc., Waltham, MA, USA), and the supernatants were filtered through a 0.22 μm nylon membrane (Pall Corp., New York, NY, USA).

The presence of phages was determined using a spot assay on a soft agar bacterial lawn. Briefly, serial dilutions of the phage-containing supernatant (10^5^ to 10^8^ PFU/mL) were prepared. A bacterial lawn was first established by mixing 100 µL of an overnight culture of the host strain with 3 mL of soft agar (TSB with 0.5% agar) and pouring the mixture onto TSA plates (Oxoid Ltd., Hants, UK). After solidification, 10 µL of each diluted and filtered supernatant was spotted onto the lawn and allowed to dry. Plates were then incubated at 37 °C for 18 h. The appearance of clear lysis zones (plaques) confirmed the presence of lytic phages. For purifying the selected phages, the isolated plaque was diluted in 1000 µL of SM buffer (50 mM Tris-Cl, pH 7.5, 100 mM NaCl, 8 mM MgSO_4_·7H_2_O), centrifuged at 8000× *g* for 10 min at 4 °C using a Sorvall Legend Micro17R (Thermo Fisher Scientific Inc., Waltham, MA, USA), and then filtered through an Acrodisc™ syringe filter with a 0.2 μm nylon membrane (Sigma Aldrich, Edo. de México, Mexico). This purification process was repeated at least three times. For determining the phage titer, a series of dilutions was subjected to the double-layer agar method and the phage titer was calculated by dividing the number of plaques observed by the volume of phages added, multiplied by the dilution factor, and expressed as plaque-forming units (PFU) per mL. The purified phages were stored either at 4 °C in SM buffer for immediate use or at −80 °C with 20% (*v*/*v*) glycerol for longer storage.

### 4.4. Host Range of Phage and Efficiency of Plating

The host range of the purified phage was determined using the double-layer agar method, as previously described [82] with minor modifications. Bacterial lawns of the ten clinical *A. baumannii* strains, thirteen ESKAPE pathogens, and two Gram-negative pathogens were prepared independently by combining 1 mL of the overnight bacterial cultures with 3 mL of soft agar (TSB with 0.5% agar) and pouring this mixture onto TSA agar plates. After the bacterial lawn solidified, 10 μL of diluted phage solution (10^5^ PFU/mL) was applied to each bacterial lawn. The phages were evaluated by the appearance of clear zones of bacterial lysis following overnight incubation at 37 °C. Based on the characteristics of the plaques observed, the bacteriolytic activity of the phages was categorized according to the previous study [12] with some modifications, including no lysis, phage resistance, partial lysis, nebula lysis, and productive infection. The development of clear plaques on a soft agar layer indicated a productive infection.

The twenty-two isolated phages were further evaluated for productive infection using the efficiency of plating (EOP) method evaluated by Mirzaei et al. [83] with some modifications. Each phage was tested in triplicate at two dilutions (10^5^ and 10^6^ PFU/mL) against *A. baumannii* strain AbAK04 which had previously shown lysis in spot assays. The testing conditions were the same as those used in the spot assays, using bacterial cultures in the stationary growth phase and the double-layer agar method. In brief, the bacterial strains were incubated for 18 h at 37 °C. Subsequently, 1000 µL of each bacterial culture was combined with 100 µL of diluted phage lysate and used in double-layer agar assays. After overnight incubation at 37 °C, the number of PFU was counted for each combination. The EOP was calculated by dividing the average PFU in the target bacteria by the average PFU in the host bacteria. Based on the EOP values, the strains were classified into the following categories: “high efficiency” (EOP ≥ 0.5), “medium efficiency” (0.1 ≤ EOP < 0.5), “low efficiency” (0.001 < EOP < 0.1), and “inefficient” (EOP ≤ 0.001).

### 4.5. Phage DNA Extraction, Sequencing and Genome Assembly, Detection of Lysogeny

Phage genomic DNA was extracted using the phenol–chloroform technique as described by Sambrook & Russell [84]. One mL of the phage suspension was treated with 10 μL of DNase I/RNase A (10 mg/mL) at 37 °C for 30 min, followed by the addition of 50 μL of SDS (10%), 40 μL of EDTA (0.5 M), and 2.5 μL of proteinase K (20 mg/mL) and incubation at 56 °C for 2 h. An equal volume of phenol–chloroform (1:1, *v*/*v*) was added, and the mixture was centrifuged at 3500× *g* for 10 min. The recovered aqueous layer was carefully transferred and mixed with another equal volume of phenol–chloroform (1:1, *v*/*v*), and this process was repeated three more times. After centrifugation at 3500× *g* for 10 min, the final aqueous phase was combined with 200 μL of 3 M sodium acetate and ethanol, and the sample was placed at −20 °C overnight before being centrifuged at 15,000× *g* for 30 min. The DNA pellet was washed with 70% ethanol, air dried, dissolved in 20 μL of nuclease-free water, and stored at −20 °C. The final DNA concentration and quality were measured with a NanoDrop 2000c spectrometer (Thermo Scientific, Wilmington, NC, USA), and were assessed by gel electrophoresis, respectively.

For sequencing the phage genomes, DNA libraries were constructed following the manufacturer’s protocol with the Nextera XT Library Preparation Kit (Illumina, San Diego, CA, USA). After the library preparation, DNA quantification was performed using a Qubit 2.0 fluorometer (Thermo Fisher Scientific, Waltham, MA, USA), and the phage genome sequencing was carried out using the Illumina MiniSeq System at CIAD Mazatlán (Centro de Investigación en Alimentación y Desarrollo, Mazatlán Unit) with a 2 × 150 bp paired-end approach over 300 cycles. The raw sequencing data were processed for quality control using fastp version 0.23.0 [85], and the assembly of the genome was conducted using SPAdes version 3.15.5 [86], applying a coverage threshold of 10x to construct the contigs.

### 4.6. Bioinformatics Analysis

Contigs were annotated using the PHANNOTATE algorithm within PATRIC version 3.36.16.1 (available online: https://www.patricbrc.org/app/Annotatio; 8 August 2024). Phage identification was performed using BLASTN to compare closely related reference sequences in the NCBI database. These reference genomes sequence alignments were performed to the isolated phages with Geneious version 9.1.8 (available online: https://www.geneious.com/; 29 January 2025). Phage lifestyles were classified with PhageAI version 0.10.0 software (available online: https://phage.ai/; 3 February 2025). Virulence and antibiotic resistance genes were annotated using ABRIcate version 0.8.13 (available online: https://github.com/tseemann/abricate; 17 February 2025), from databases such as NCBI, CARD, ARG-ANNOT, Resfinder, MEGARES, and VFDB. tRNA genes were predicted using tRNAscan-SE version 2.0 [87]. Open reading frames (ORFs) were identified using the ORF search tool (available online: https://www.ncbi.nlm.nih.gov/orffinder/; 19 February 2025) and protein functions were analyzed with BLASTp on the NCBI server (available online: https://blast.ncbi.nlm.nih.gov/Blast.cgi; 21 February 2025), using a non-redundant protein database with a score threshold >50 and an *e*-value <1.0 × 10^−3^. The characteristics of the ORFs were further analyzed using Artemis Comparison Tool version 18.2.0. [88] and DNAPlotter version 18.2.0 [89], respectively. For the phylogenetic analysis, alignments were compiled using the VICTOR platform (available online: https://ggdc.dsmz.de/victor.php; 24 February 2025) to explore phylogenetic relationships by creating phylogenomic trees using the Genome-BLAST remote phylogeny method. The results in the Newick format were then used to construct a phylogenetic tree with iTOL version 6.9.1 (available online: http://itol.embl.de; 13 March 2025). Intergenomic similarities between viral genomes were assessed using VIRIDIC software (https://rhea.icbm.uni-oldenburg.de/viridic/; accessed 8 April 2025) with standard BLASTN settings. Additionally, the clinker pipeline, version 0.0.28 [90] was used to perform a comparative analysis of the phages when compared to their reference genomes. For the identification of ORF62, which encodes the tailspike endosialidase, the analysis was performed using InterProScan (available online: https://www.ebi.ac.uk/interpro/search/sequence/; 27 April 2025) [22].

### 4.7. Phage-Mediated Antibacterial Activity Assays

In vitro antibacterial activity was evaluated using a 96-well microplate format using bacterial cultures in the stationary growth phase (10^8^ CFU/mL) and phage suspensions at a multiplicity of infection (MOI) of 10, 1, and 0.1 [91]. The positive control consisted of 200 μL of bacterial culture, and the negative control was 200 μL of TSB. For each experimental condition, 100 μL of TSB and 80 μL of the phage dilution were mixed to achieve the desired MOI, followed by the addition of 20 µL of the bacterial culture in a final volume of 200 µL per well. The microplate was incubated at 37 °C for 17 h using a Synergy HT microplate reader (Biotek, Inc., Winooski, VT, USA), and absorbance measurements were taken at a wavelength of 600 nm every 15 min after orbital shaking before each measurement. The lysogenic behavior of a phage was verified by several experimental tests, especially when no genes associated with lysogeny are detected in the phage genome and it is classified as temperate by some bioinformatics program. The lysogenic activity of the isolated phages was detected in triplicate, according to a study described previously [26].

For performing the one-step growth curve, the burst size was determined from triplicate experiments by calculating the ratio of the final count of released phage particles to the initial count of infected bacterial cells, divided by the number of infected cells (phage titer at 0 min—phage titer at 0 min with chloroform), as in previous studies [48]. The adsorption test was performed in triplicate using a modified version of the method described by Park and Park [92]. In detail, bacterial cultures (1 mL) in the stationary growth phase (10^8^ CFU/mL) were subjected to centrifugation at 8000× *g* for 10 min, resuspended in TSB and further diluted (10^7^ CFU/mL) in 9 mL of fresh TSB. The bacterial suspension was then mixed with 1 mL of the phage solution (MOI = 0.1) and further incubated at 37 °C for 5 min. After incubation, the samples were centrifuged at 15,000× *g* for 1 min to eliminate free unabsorbed phages, and the phage titer was calculated by the double-layer agar method, which corresponded to time point 0. The procedure above was repeated every 5 min for a total of 20 min. The percentage of free phages was evaluated by calculating the number of phages at a specific time divided by the initial number of phages multiplied by 100.

### 4.8. Statistical Analysis

All statistical analyses were performed using GraphPad Prism 8.0.1 (GraphPad Software, Inc., La Jolla, CA, USA) and Minitab^®^ 19, Minitab Statistical Software (Minitab, LLC., State College, PA, USA). Quantitative values were reported as the mean ± standard deviation. For the bacteriolytic activity assay, a repeated-measures design was utilized by defining the MOI as a nested factor and time as a crossed factor. Group comparisons were then conducted using Tukey’s post-hoc test. Probability values less than 0.05 were considered significant for all statistical analyses.

## Figures and Tables

**Table 1:** Summary of characteristics of the different *Acinetobacter baumannii*-specific phages.

Host Range (Spot Test)/Efficiency of Plating (EOP) ^a^																										Plaque Diameter (mm) ^c^
Phage Strain ID	*Acinetobacter baumannii* Strain										ESKAPE Pathogens													GNPs ^b^		
	AbAK01	AbAK02	AbAK03	AbAK04	AbAK05	AbAK06	AbAK07	AbAK08	AbAK09	AbAK10	PaAK07	PaAK08	PaAK10	PaAK11	KpAK11	KpAK13	KpAK14	KpAK15	KpAK16	KpAK17	KpAK18	EfAK11	SaAK16	EcoAK05	EcoAK06	
AKO8a	-	-	-	+++ (1)H	-	-	-	-	-	-	-	-	-	-	-	-	-	-	-	-	-	-	-	-	-	1
89P13	-	-	-	+++ (1)H	-	-	-	-	-	-	-	-	-	-	-	-	-	-	-	-	-	-	-	-	-	1
κeane	-	-	-	+++ (1)H	-	-	-	-	-	-	-	-	-	-	-	-	-	-	-	-	-	-	-	-	-	1
C∞ldPlαy	-	-	-	+++ (1)H	-	-	-	-	-	-	-	-	-	-	-	-	-	-	-	-	-	-	-	-	-	1
MCR	-	-	-	+++ (0.7)H	-	-	-	-	-	-	-	-	-	-	-	-	-	-	-	-	-	-	-	-	-	1
PS118	-	-	-	+++ (1)H	-	-	-	-	-	-	-	-	-	-	-	-	-	-	-	-	-	-	-	-	-	2
KOL	-	-	-	+++ (0.4)M	-	-	-	-	-	-	-	-	-	-	-	-	-	-	-	-	-	-	-	-	-	1
B612	-	-	-	+++ (1)H	-	-	-	-	-	-	-	-	-	-	-	-	-	-	-	-	-	-	-	-	-	1
Green-8	-	-	-	+++ (0.1)L	-	-	-	-	-	-	-	-	-	-	-	-	-	-	-	-	-	-	-	-	-	1
PB14N	-	-	-	+++ (1)H	-	-	-	-	-	-	-	-	-	-	-	-	-	-	-	-	-	-	-	-	-	1
DA78	-	-	-	+++ (1)H	-	-	-	-	-	-	-	-	-	-	-	-	-	-	-	-	-	-	-	-	-	1
TAM10	-	-	-	+++ (1)H	-	-	-	-	-	-	-	-	-	-	-	-	-	-	-	-	-	-	-	-	-	1
IDQ7	-	-	-	+++ (1)H	-	-	-	-	-	-	-	-	-	-	-	-	-	-	-	-	-	-	-	-	-	2
PN1412	-	-	-	+++ (1)H	-	-	-	-	-	-	-	-	-	-	-	-	-	-	-	-	-	-	-	-	-	2
CRL20	-	-	-	+++ (1)H	-	-	-	-	-	-	-	-	-	-	-	-	-	-	-	-	-	-	-	-	-	1
MRL18	-	-	-	+++ (1)H	-	-	-	-	-	-	-	-	-	-	-	-	-	-	-	-	-	-	-	-	-	2
CIM23	-	-	-	+++ (1)H	-	-	-	-	-	-	-	-	-	-	-	-	-	-	-	-	-	-	-	-	-	1
AM	-	-	-	+++ (1)H	-	-	-	-	-	-	-	-	-	-	-	-	-	-	-	-	-	-	-	-	-	1
Pyro	-	-	-	+++ (0.4)M	-	-	-	-	-	-	-	-	-	-	-	-	-	-	-	-	-	-	-	-	-	1
dakota	-	-	-	-	-	-	-	-	-	-	-	-	-	-	-	-	-	-	-	-	-	-	-	-	-	1
Août	-	-	-	+++ (0.4)M	-	-	-	-	-	-	-	-	-	-	-	-	-	-	-	-	-	-	-	-	-	1
Δspirit	-	-	-	+++ (1)H	-	-	-	-	-	-	-	-	-	-	-	-	-	-	-	-	-	-	-	-	-	1

^a^ Efficiency of plating (EOP) was classified as “H” high efficiency (EOP ≥ 0.5), “M” medium efficiency (0.1≤EOP < 0.5), “L” low efficiency (0.001 < EOP < 0.1) and “I” ineffective (EOP ≤ 0.001); Host range results were classified as productive infection (+++), and no lysis (-). ^b^ GNPs represents Gram-negative pathogen. ^c^ Plaque diameter obtained from the double agar technique against respective host.

**Table 2:** Summary of the genomic characteristics of phages.

Phage Common Name	Phage Taxonomy Name	Genome Length (bp)	GC Content (%)	Total No. of ORFs ^a^	No. of ORFs with Known Functions	tRNAs ^b^	Antibiotic Resistance Genes	Virulence Genes	Lysogeny Genes
CIM23	vB_AbaS_CIM23	44,512	50.6	145	35	0	0	0	1
B612	vB_AbaS_B612	44,702	50.4	146	34	0	0	0	1
89P13	vB_AbaS_89P13	43,176	50.4	141	35	0	0	0	1
AKO8a	vB_AbaS_AKO8a	67,450	49.9	157	149	0	0	0	0
PS118	vB_AbaS_PS118	46,430	50.5	161	38	0	0	0	1
MCR	vB_AbaS_MCR	45,205	50.4	139	34	0	0	0	0
CRL20	vB_AbaS_CRL20	45,335	50.2	147	39	0	0	0	0
IDQ7	vB_AbaS_IDQ7	44,703	50.3	148	38	0	0	0	1

^a^ ORFs represent open reading frames identified within the genome, which may encode functional proteins. ^b^ tRNAs represent transfer RNAs identified in the genome, which play a crucial role in protein synthesis by delivering amino acids to the ribosome.

## Data Availability

The complete genome sequence of vB_AbaS_PS118, vB_AbaS_MCR, vB_AbaS_IDQ7, vB_AbaS_CRL20, vB_AbaS_CIM23, vB_AbaS_B612, vB_AbaS_AKO8a, and vB_AbaS_89P13 have been submitted to the GenBank database and the accession numbers PQ997946, PQ997947, PQ997948, PQ997949, PQ997950, PQ997951, PQ997952, and PQ997953 has been assigned, respectively.

## Supplementary Materials

The supporting information can be downloaded at: https://www.mdpi.com/article/10.3390/ijms26136141/s1.
