# Supplementary material for: Genomic and Functional Characterization of Novel Phages Targeting Multidrug-Resistant Acinetobacter baumannii
Source: Int J Mol Sci. 2025 Jun 26;26(13):6141. doi: 10.3390/ijms26136141 (PMC12249539; doi:10.3390/ijms26136141)
Supplement: Supplementary file 1 [file ijms-26-06141-s001.zip › ijms-3687649-supplementary.pdf]

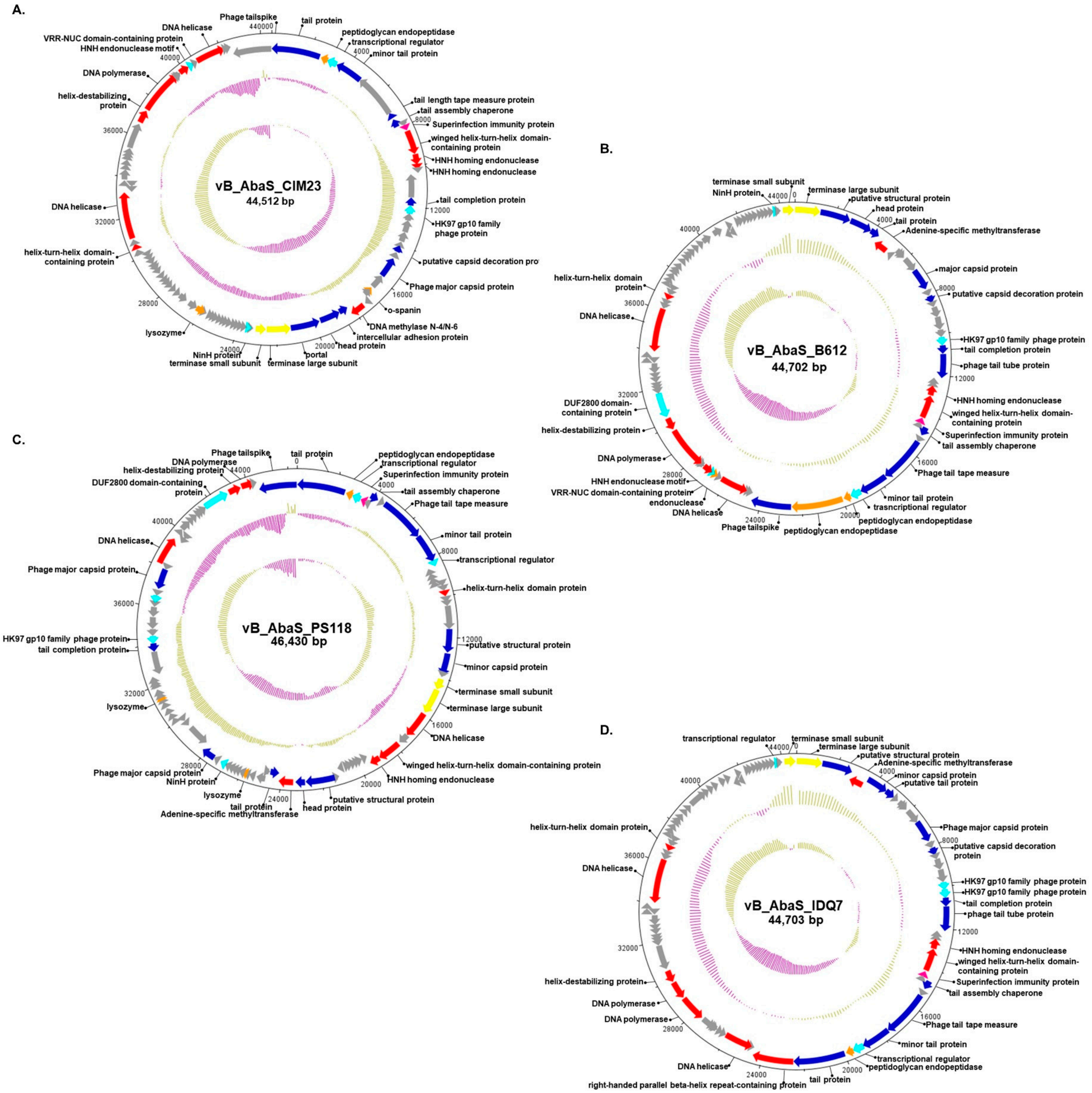

Figure S1. Genome map using DNAPlotter. A: Phage CIM23. B: Phage B612. C: Phage PS118. D: Phage IDQ7.

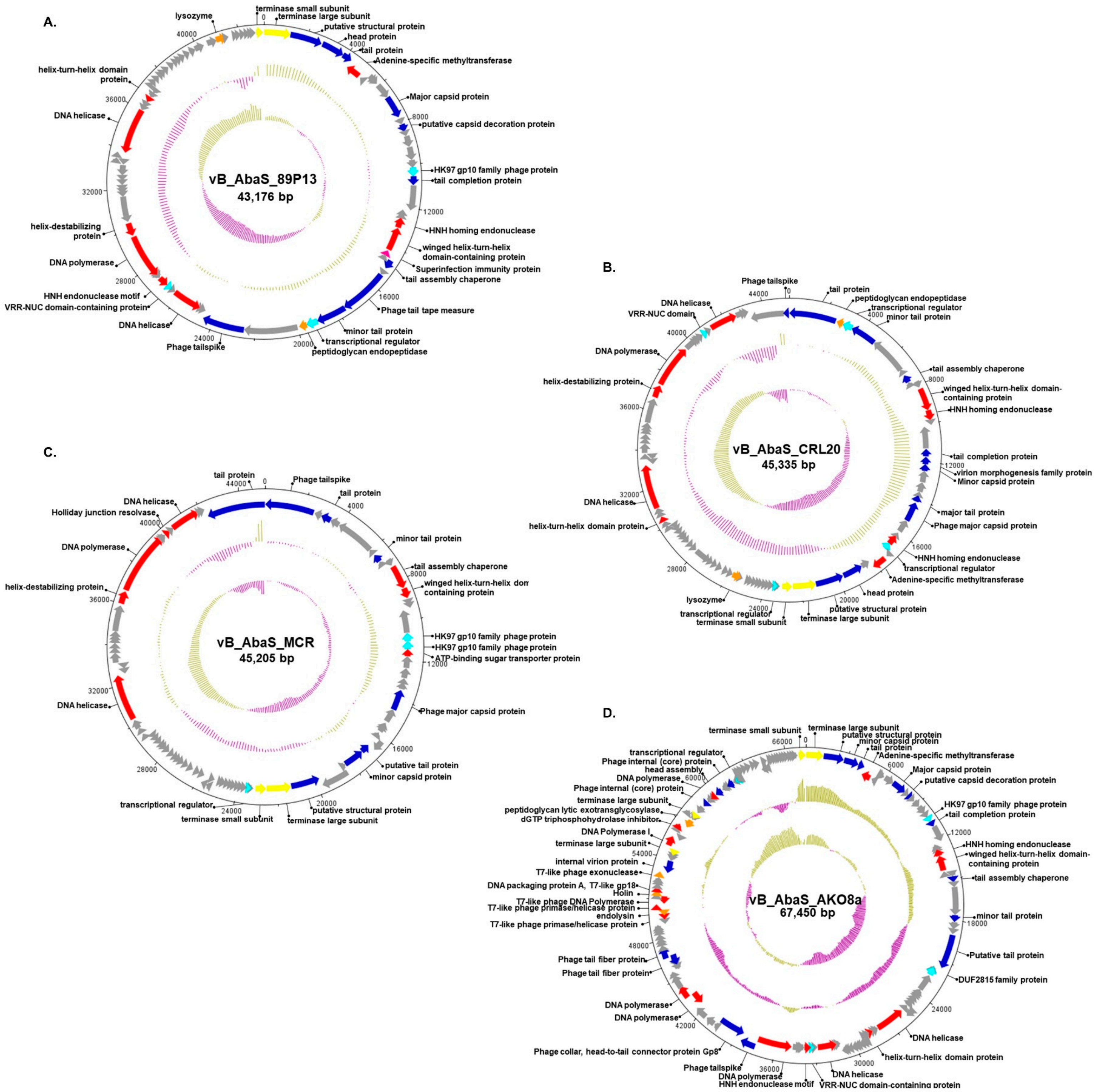

Figure S2. Genome map using DNAPlotter. A: Phage 89P13. B: Phage CRL20. C: Phage MCR. D: Phage AKO8a.

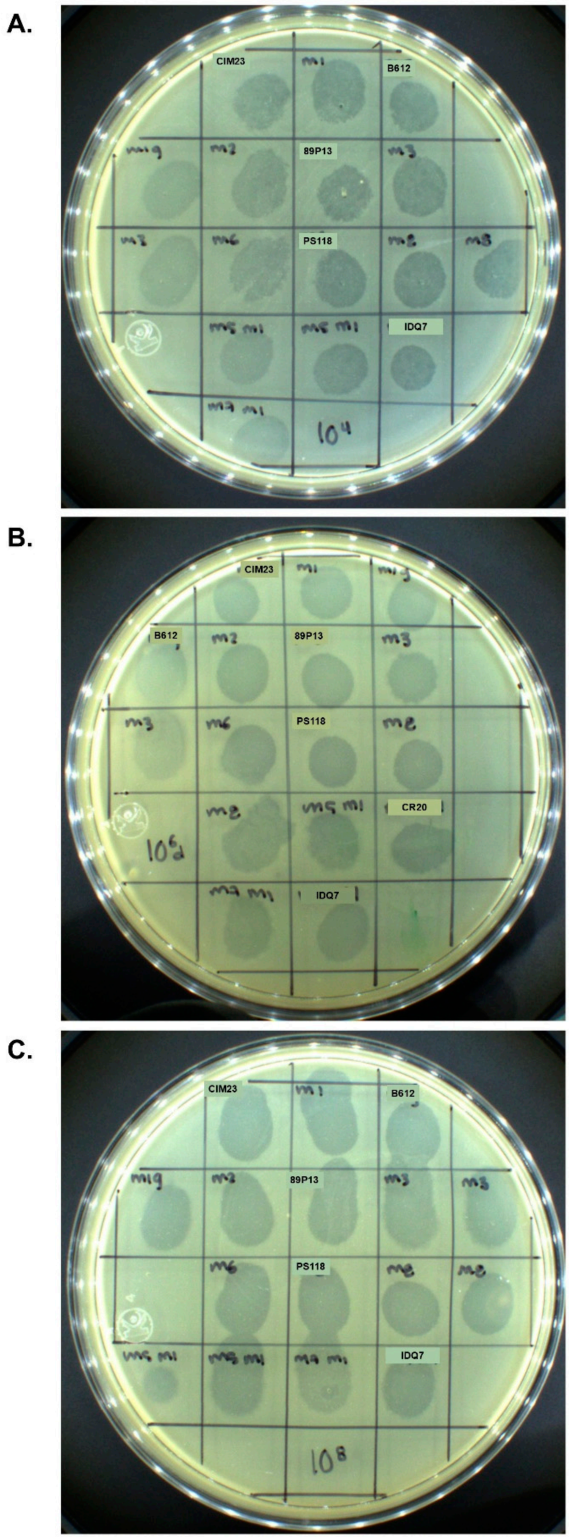

Figure S3. Attempt to generate *A. baumannii* AbAK04 lysogens. Phages PS118, B612, IDQ7, 89P13, and CIM23 failed to produce lysogenic "mesas" at the tested titers: A. 10^4; B. 10^6; and C. 10^8 PFU, after 96 h of co-incubation with *A. baumannii* strain AbAK04.

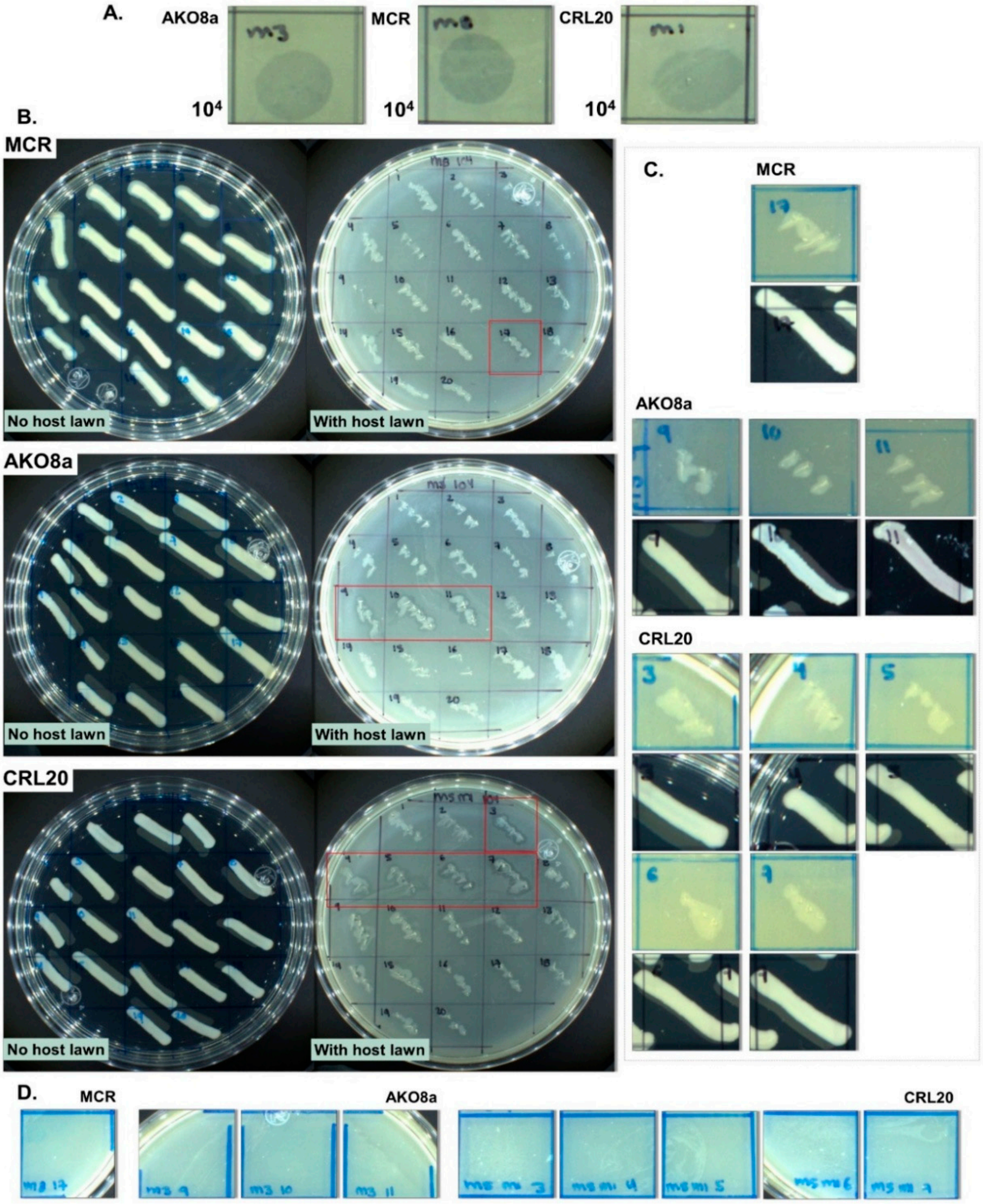

Figure S4. Attempt to produce *A. baumannii* strain AbAK04 lysogens. A: Spot test at a concentration of 10^4 PFU of phages AKO8a, MCR, and CRL20 after 96 h of incubation. B: Patch assay. Bacterial growth scraped from the synthesis zones was streaked, and individual colonies were patched on TSA plates and TSA plates overlaid with AbAK04. Red boxes indicate bacterial growth with surrounding synthesis, suggesting the presence of the corresponding phage, possibly released from a lysogen. C: After three rounds of purification of each colony, the patch assay was repeated with no indication of phage presence. D: Spot test performed with the filtered supernatant from the cultures of the colonies, with no indication of spontaneous phage release.
